# LGR5 is a conserved marker of hair follicle stem cells in multiple species and is present early and throughout follicle morphogenesis

**DOI:** 10.1038/s41598-022-13056-w

**Published:** 2022-06-01

**Authors:** Kathryn M. Polkoff, Nithin K. Gupta, Adrian J. Green, Yanet Murphy, Jaewook Chung, Katherine L. Gleason, Sean G. Simpson, Derek M. Walker, Bruce Collins, Jorge A. Piedrahita

**Affiliations:** 1 Molecular Biomedical Sciences, North Carolina State University College of Veterinary Medicine, Raleigh, NC USA; 2 North Carolina State University Comparative Medicine Institute, Raleigh, NC USA; 3 Campbell University School of Osteoplastic Medicine, Lillington, NC USA; 4 Department of Biological Sciences, North Carolina State University College of Sciences, Raleigh, NC USA; 5 Department of Animal and Avian Sciences, University of Maryland, College Park, Maryland USA; 6 RenOVAte Biosciences, Inc., Reisterstown, MD USA

**Keywords:** Adult stem cells, Skin stem cells, Genetic markers

## Abstract

Hair follicle stem cells are key for driving growth and homeostasis of the hair follicle niche, have remarkable regenerative capacity throughout hair cycling, and display fate plasticity during cutaneous wound healing. Due to the need for a transgenic reporter, essentially all observations related to LGR5-expressing hair follicle stem cells have been generated using transgenic mice, which have significant differences in anatomy and physiology from the human. Using a transgenic pig model, a widely accepted model for human skin and human skin repair, we demonstrate that LGR5 is a marker of hair follicle stem cells across species in homeostasis and development. We also report the strong similarities and important differences in expression patterns, gene expression profiles, and developmental processes between species. This information is important for understanding the fundamental differences and similarities across species, and ultimately improving human hair follicle regeneration, cutaneous wound healing, and skin cancer treatment.

## Introduction

The skin is the largest organ in the body and is responsible for maintaining homeostatic conditions such as thermoregulation, hydration, and protection from the environment^1^. To maintain these processes, the skin epidermis contains multiple populations of stem cells. One of these populations, hair follicle stem cells (HFSC), has been studied extensively in mice for understanding stem cell behavior in homeostasis and repair^2,3^. Although studies in mice have provided in depth mechanistic insight, translational studies using mouse models are limited due to differences in anatomy and physiology; mice have dense fur coats which undergo cyclic periods of growth and rest, thin dermis and epidermis, and loose skin attachment^1,4^. In contrast, humans have sparse hair coats, asynchronous follicle cycling, thick dermis and epidermis, and tight skin attachments; all these characteristics are shared with pigs, making them a widely accepted model for human skin and repair^5^. However, while several genes have been proposed as HFSC markers, they often vary across species^6,7^, making it difficult to perform functional comparisons between species.

In mice, HFSC have been defined by the leucine-rich G protein coupled receptor-5 (LGR5)^8–10^, a known potentiator of WNT signaling when bound to its ligand R-spondin^11^. Exciting findings from murine studies show that LGR5-expressing HFSC contribute to all regions of the hair follicle, including sebocytes^8^, are vital for telogen to anagen transition during hair cycling^12–14^, migrate from the hair follicle niche to contribute to re-epithelialization during wound healing^9,15^, and are the cells responsible for hair regeneration in wound-induced follicle neogenesis^16,17^. While LGR5 is a widely used marker in mice, there have been no previous studies to our knowledge that evaluate the location of LGR5 expression in the skin of humans or non-murine models. This is possibly because its study has been limited due to the lack of a reliable commercially available anti-LGR5 antibody^18,19^.

To solve this issue, we developed a transgenic pig that expresses *LGR5*-driven H2B-GFP and here we use it to identify conserved pathways of gene expression, compare cell behavior, and study the role of LGR5-expressing HFSC in fetal and post-natal stages across species.

## Results

### Generation of a porcine LGR5-H2B-GFP model

**Figure 1:**
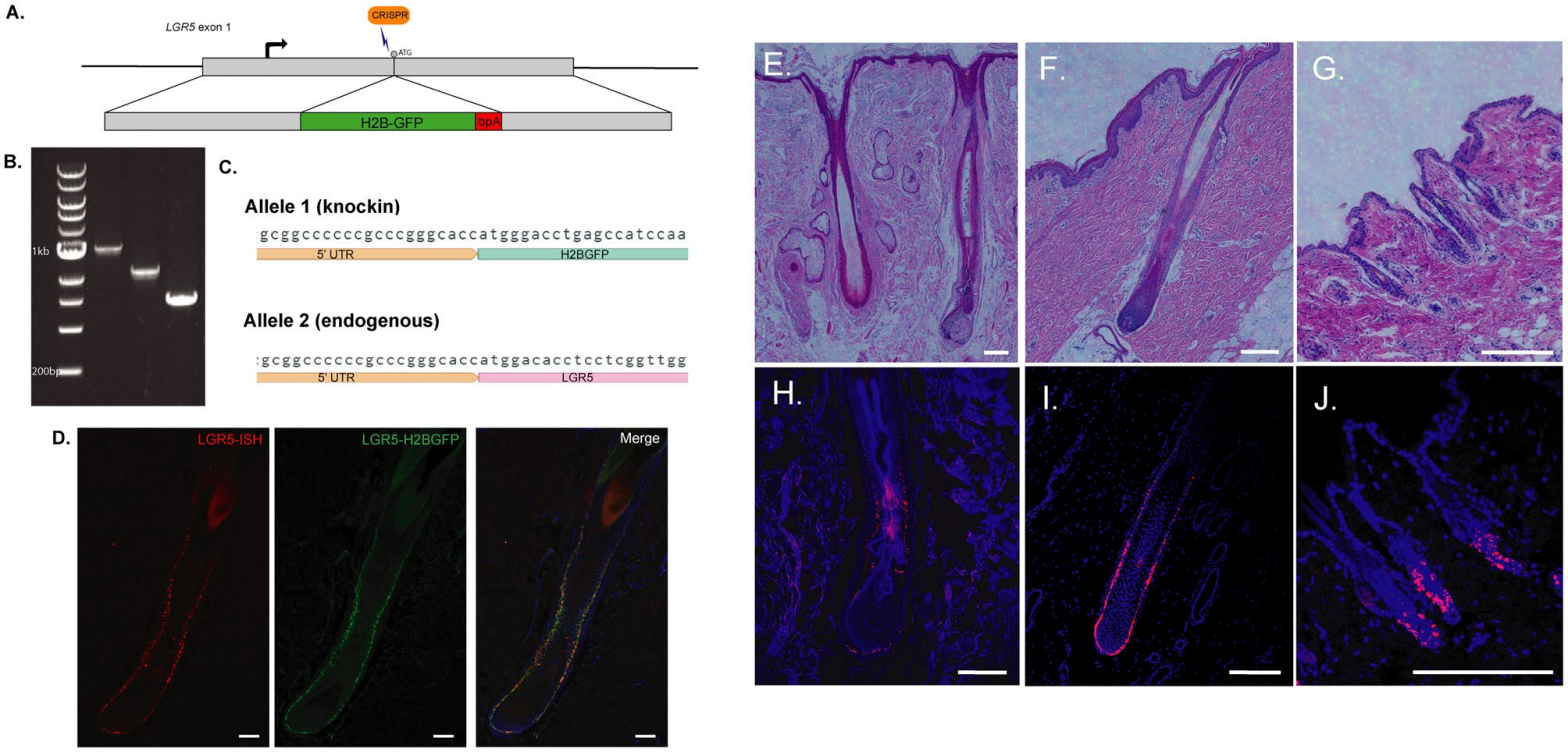
LGR5 is expressed in the hair follicle outer root sheath in the lower bulge in humans, pigs and mice. (**A**) Schematic of H2B-GFP knock-in at the porcine *LGR5* locus. 500 base pair regions of homology on either side of the CRISPR target site at the start codon were included in the homology directed repair template. (**B**) PCR amplification of DNA from a colony containing the knock-in, from left: 200 bp DNA Ladder, 5′ junction (allele #1), 3′ junction (allele #1), unedited genomic allele (allele #2) (full gel in Fig. S1). (**C**) Sequencing of *LGR5* allele #1 and allele #2 shows the endogenous locus of allele #1 remains unaltered and expresses the LGR5 protein, while allele #2 encodes H2B-GFP instead. (**D**) *LGR5* transcript expression detected by fluorescent in situ hybridization to a pig *LGR5* probe (*LGR5*-ISH) correlates with LGR5-H2BGFP expression in the skin of *LGR5-H2B-GFP* pigs. (**E**–**J**) Hair follicle structure and *LGR5* mRNA localization (RNAscope) in follicles from humans (**E**, **H**), pigs (**F**, **I**) and mice (**G**, **J**). Scale bar represents 200 μM.

To facilitate the study of LGR5-expressing cells in a non-murine species, we generated a transgenic porcine model expressing H2B-GFP under the control of the *LGR5* promoter using CRISPR/Cas9 mediated gene knock-in. We elected to use a nuclear H2B-GFP we and others have previously used^2,20,21^. The H2B-GFP sequence was inserted into exon 1 of one *LGR5* allele and after validation for accurate transgene insertion by PCR and sequencing (Fig. 1A–C), clonal cell lines were used for somatic cell nuclear transfer. To examine whether H2BGFP expression accurately reflects *LGR5* mRNA expression, we overlaid RNA fluorescent in situ hybridization (RNA-FISH) of porcine *LGR5* mRNA and LGR5-H2BGFP protein expression (Fig. 1D). Based on colocalization, H2BGFP in this model is faithfully representative of *LGR5* expression.

### LGR5 is a marker of the outer root sheath and bulge cells in all stages of the hair cycle in human, porcine, and mouse skin, and is expressed at a low level in the inner root sheath

Studies in mice have shown LGR5 expression in the lower bulge and outer-root sheath throughout anagen, catagen and telogen. The “bulge” niche is defined as the region in the hair follicle below the isthmus and infundibulum at the point where the arrector pili muscle attaches to the hair follicle (Fig. 1E–G)^22^. Our results show that all three species express LGR5 in the bulge region of the hair follicle (Fig. 1H–J). In anagen, in the porcine, this expression extends from the bulge region down through the outer root sheath all the way to the base of the hair follicle (Fig. S1A–C). In catagen and telogen, fewer cells are marked by LGR5 and expression is confined to the bulge region, and importantly LGR5-H2BGFP cells maintain contact with the dermal papillae (Fig. S1). RNA-FISH of human arm skin shows the same pattern of *LGR5* expression in human hair follicles as in porcine follicles throughout the hair cycle (Fig. S1D–F).

In addition, a closer examination of nuclear GFP fluorescence in the pig beyond the outer root sheath reveals low or absent LGR5 mRNA (RNA-ISH Fig. S1G–I) and a GFP-dim population in the inner root sheath (Fig. S2A). This LGR5-low population is expressed in the inner root sheath above and at the bulge (Fig. S2B–D).

### LGR5-expressing cells in the hair germ give rise to the inner root sheath and hair shaft

**Figure 2:**
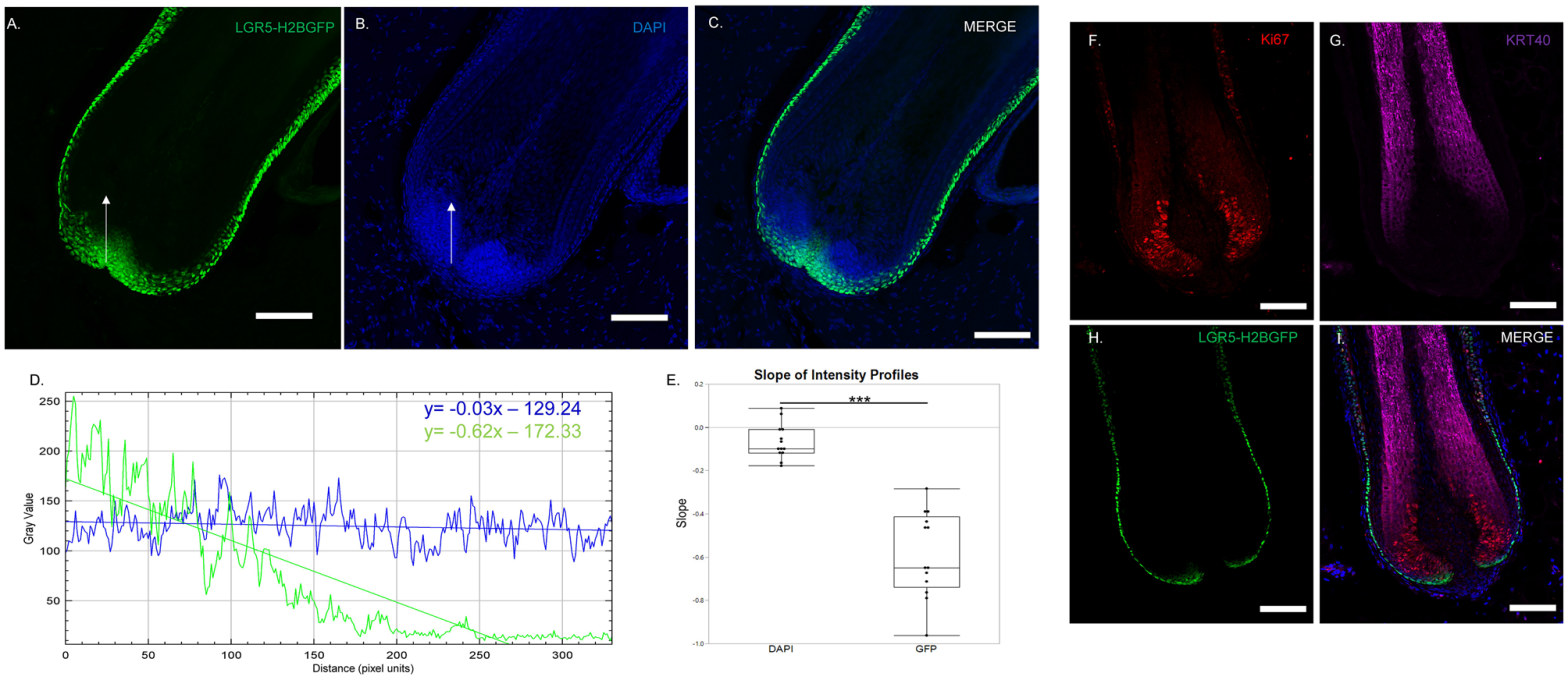
LGR5-H2BGFP cells give rise to transit amplifying populations in the hair matrix in anagen. (**A**–**C**) 20 × magnification confocal image of base of anagen hair follicle depicting LGR5-H2BGFP expression as compared with DAPI. Intensity profile of each fluorescence along the white arrow is plotted in (**D**), which is summarized by the fit line. (**E**) Quantification of slope from profile plots of GFP or DAPI profiles, with profile line drawn from base toward inner root sheath, showing that LGR5-H2BGFP intensity is diluted significantly while DAPI remains relatively unchanged, n = 12 follicles from 3 different pigs*.* IHC Staining to detect (**F**) KI67 (**G**) Keratin 40 (KRT40) or (**H**) LGR5-H2BGFP signal dilution as LGR5 cells give rise to the proliferating transit amplifying cells (KI67). This population then differentiating into the inner root sheath or hair shaft (KRT40). (**I**) Merge. Scale bar represents 100 μM.

During anagen, HFSC are activated, exit the bulge region, proliferate downward in the outer root sheath to the hair matrix region. Here, they give rise to a transit amplifying population, which ultimately differentiates into 7 different lineages within the hair shaft and inner root sheath^23–25^. Thus, we next asked whether LGR5-H2BGFP cells in the porcine hair follicle ultimately give rise to the hair and the inner-root sheath. Here, we found three distinct phases: (1) The LGR5-H2BGFP cells in contact with the basement membrane at the base of the follicle, (2) the transit amplifying population marked by KI67 and (3) the differentiated hair shaft cells marked by KRT 40.The H2BGFP signal in the nucleus of LGR5 expressing cells allows limited lineage tracing as the H2BGFP intensity drops by dilution with wildtype H2B with each cell division^2,26^. LGR5-expressing cells at the base of the hair follicle in contact with the basement membrane and adjacent to the dermal papilla, show bright GFP expression, which decreases drastically in intensity as the cells divide to generate the transit amplifying population (Fig. 2A–C). The line profile drop in intensity was compared to DAPI, which remains constant along the same vector (Fig. 2D, E). KI67 staining confirmed that the LGR5-H2BGFP cells are dividing as the GFP intensity decreases (Fig. 2F), and that the LGR5-/KI67+ transit amplifying cell populations terminally differentiate into the inner root sheath and hair shaft and cortex, as shown by expression of hair keratin, KRT 40 (Fig. 2G–I).

### Immunohistochemistry and gene expression analysis of LGR5-H2BGFP cell localization with known markers of the epidermis

**Figure 3:**
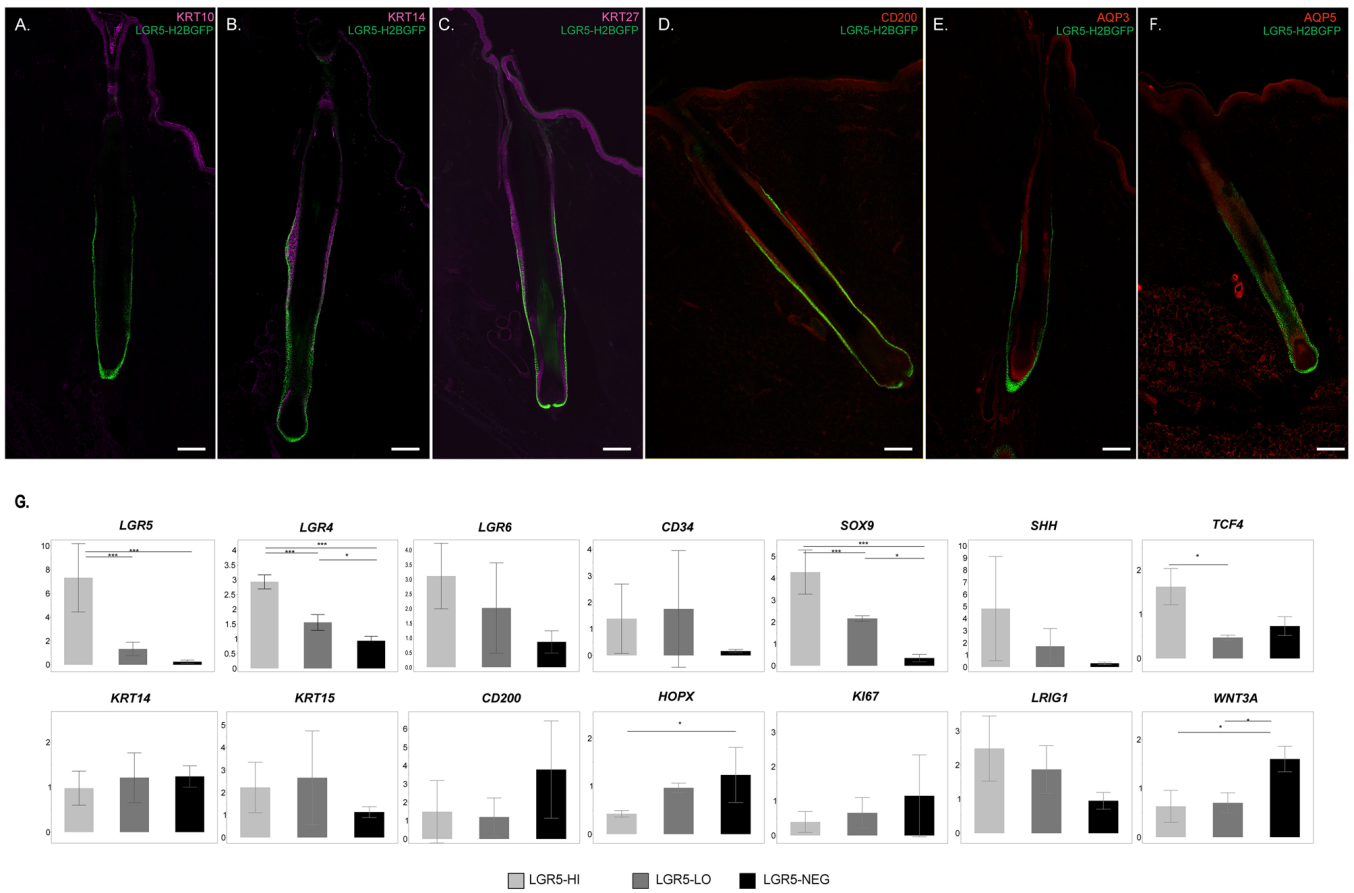
LGR5-H2BGFP epidermal cells express known stem cell and epidermal markers. Confocal microscopy images of hair follicles following antibody staining with anti-GFP and known skin markers and overlaid with DAPI. (**A**) Keratin 10 (KRT10), (**B**) Keratin 14 (KRT14), (**C**) Keratin 27 (KRT27), (**D**) CD200, (**E**) Aquaporin 3 (AQP3), (**F**) Aquaporin 5 (AQP5). Scale bar represents 200 μM. (**G**) Relative expression of known stem cell markers based on delta-delta Ct analysis of RT-qPCR from LGR5-H2BGFP-high (LGR5-HI, light gray), LGR5-H2BGFP-low (LGR5-LO, dark gray), or LGR5-H2BGFP negative (LGR5-NEG, black) populations after flow sorting. Each sample is normalized to *ACTB* and *GAPDH* and then to unsorted epidermis, n = 3 pigs. ANOVA followed by Tukey’s honestly significant difference (HDS) test ***P < 0.005, *P < 0.05, error bars indicate standard deviation.

Next, we applied antibody staining to evaluate LGR5 localization in the context of well-known epidermal markers of mice and/or human skin. KRT10, a marker of differentiated interfollicular epidermis cells, does not co-localize with LGR5-H2BGFP, and was not detected in the follicle below the isthmus (Fig. 3A). KRT14 (Fig. 3B), which forms an intracellular dimer with KRT5^27^, is present in the entire basal layer of the porcine epidermis, and co-localize with LGR5-H2BGFP in the bulge region of the hair follicle, but not the base. KRT27, a marker of the Henle, Huxley and Cuticle regions of the inner root sheath^28^, stains the LGR5-low populations in the inner root sheath, in addition to the entire interfollicular epidermis (Fig. 3C). Unlike it’s reported stem-cell specific affinity in humans^29^, CD200 stained all layers of the epidermis in pigs, including LGR5-H2BGFP cells in the bulge (Fig. 3D). Aquaporin-3 (AQP3), a known marker of differentiated keratinocytes^30,31^, shows minimal co-localization with LGR5-H2BGFP, and instead stains the interfollicular epidermis and continues through the inner root sheath (Fig. 3E). Aquaporin-5 (AQP5) has recently been suggested as an alternative to LGR5 to mark stem cells in the stomach^18^, but is not specific in the skin (Fig. 3F). From this we can confirm that in the pig, LGR5-H2BGFP HFSC partially co-localize with markers of the basal layer stem cells including KRT14, CD200 and AQP5, but not with more differentiated markers of the epidermis and keratinocytes such as KRT10, KRT27, and AQP3.

While LGR5 is recognized as a marker of HFSC in mice^8^, other stem cell markers such as SOX9, TCF4, LRIG1, LGR4 and LGR6, have also been linked to stem cell populations of the epidermis in mice and humans^22,32^. To examine whether LGR5-H2BGFP positive cells HFSCs also express other stem cell markers, we used fluorescence activated cell sorting (FACS) to separate the LGR5-H2BGFP high, low, and negative populations (Fig. S3) and performed RT-qPCR separately on each population (Fig. 3G). Our results show that LGR5-H2BGFP cells are also enriched for *LGR4* and *LGR6* and that *SOX9, and TCF4* are significantly upregulated in the LGR5-high populations. Markers that have been used to identify and/or enrich for murine HFSC such as *KRT5, KRT15,* and *CD34* are not significantly enriched in the porcine LGR5-H2BGFP HFSC. We also confirmed the relationship between GFP signal intensity and mRNA expression in LGR5-H2BGFP high, LGR5-H2BGFP low and LGR5-H2BGFP negative cells (Fig. 3G).

### Transcriptome analyses reveal extracellular matrix and structure organization as top conserved pathways across species

**Figure 4:**
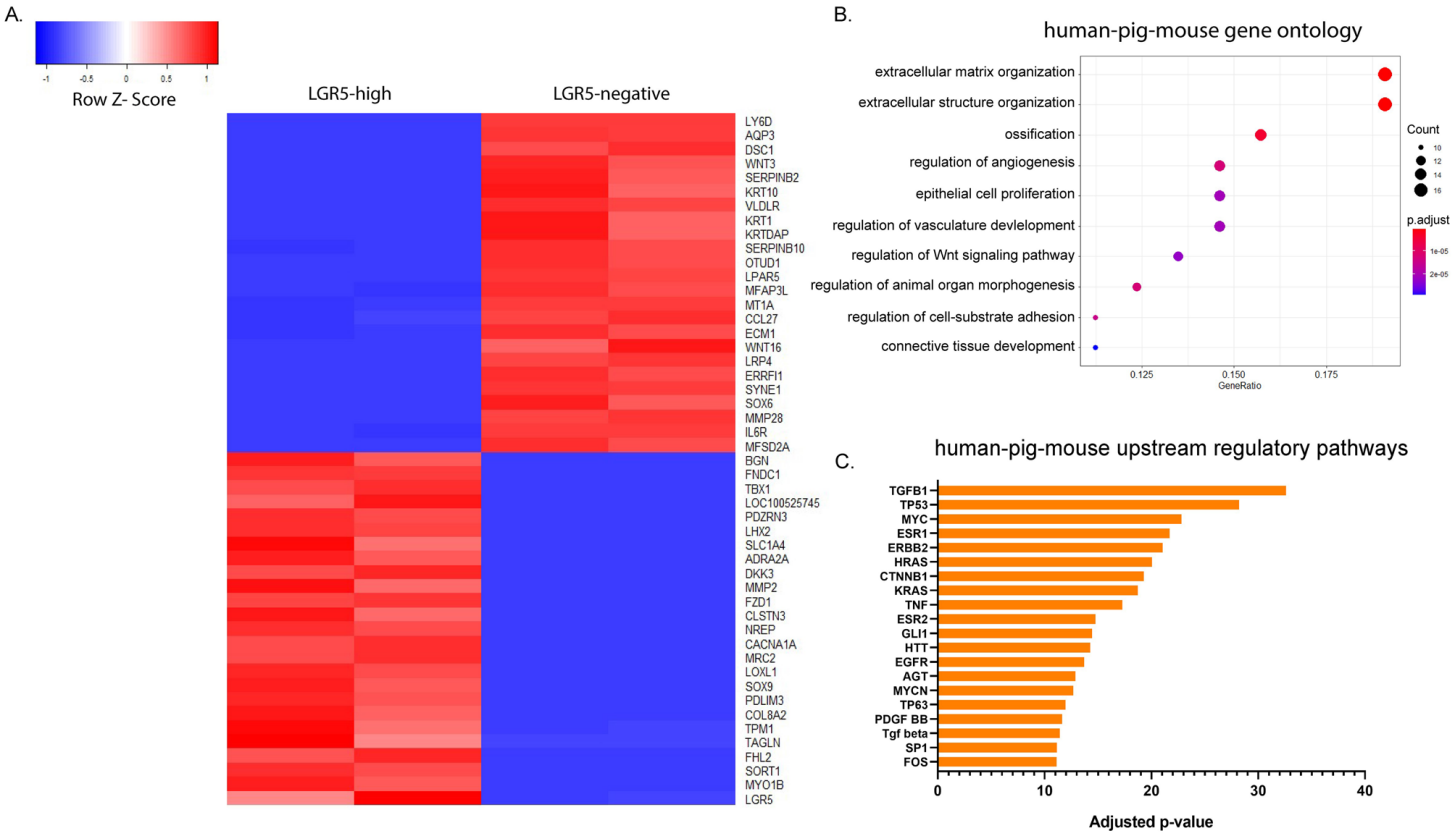
Gene ontology pathways in human, pig, and mouse LGR5-expressing epidermal populations are enriched in extracellular matrix structure and organization. (**A**) Top significantly upregulated (25) and downregulated (25) genes between LGR5-H2BGFP-high and LGR5-H2BGFP negative sorted pig samples. Bulk RNAseq samples were prepared from sorted populations from two pigs. Note confirmation of *LGR5* and *SOX9* upregulation in the LGR5-H2BGFP high population. (**B**) Comparison of all three species showing common gene ontology terms from genes that are upregulated in LGR5-high epidermis across all three species. (**C**) Upstream regulators that target a significant portion of the genes that are upregulated in LGR5 + cells from mice, human, and pig datasets. Each bar represents significance (negative log) of a set of multiple genes that impact a single regulatory pathway.

To examine the gene expression profile of LGR5-H2BGFP cells in the pig at a deeper molecular level, we performed RNA sequencing (RNAseq) on RNA extracted from FACS sorted LGR5-H2BGFP high (LGR5-high) or LGR5-H2BGFP negative (LGR5-negative) cells (as in Fig. S3). Statistical analyses found 1619 significantly upregulated and 1161 significantly downregulated genes in the LGR5-high population compared with the LGR5-negative population after correction with Bonferroni adjustment for multiple comparisons. A heatmap of the 25 most differentially upregulated and downregulated genes shows significant differences in many epidermal, structural and stem cell genes (Fig. 4A). Two of the top three most significantly down-regulated genes in the LGR5-high group were *KRT10* and *AQP3*, which are confirmed by our immunostaining results (Fig. 3A,E).

We next aimed to evaluate how the gene expression profile and signalling pathways of the LGR5-H2BGFP cells in pigs compares with the human and the mouse. To do so, we used publicly available single cell RNAseq datasets from human^33^ and mouse^34^, and clustered the cells based on high or undetectable levels of *LGR5* expression. After quality control, we retained 3593 and 1422 human and mouse cells, respectively for further analysis. Clustering based on *LGR5* expression yielded 3400 negative and 59 positive human cells and 1278 negative and 82 positive mouse cells. In the LGR5 positive cells a total of 836 genes common to all three species were identified, as well as 1,329 (human), 953 (mouse) and 5,763 (pigs) uniquely expressed genes (q-value ≤ 0.1). The gene ontology analysis revealed conserved pathways in comparisons of species by pairs, with extracellular matrix organization, extracellular structure organization and cell-substrate adhesion common to all three species (Fig. 4B and Fig. S4A–C) suggesting a role for LGR5 cells in participating in modulation of the extracellular microenvironment. Comparative analysis of all three species (human, porcine and mouse) using IPA revealed a number of potential shared upstream regulators with the 20 most significant (adjusted P value) genes shown in Fig. 4C, including those involved in growth (*TGFB family,* specifically *TGFB1*), transcription factors and cell signaling (*MYC*, *MYCN*, *CTNNB1*, *TNF*, *ESR1*, *ESR2*, *TP63*), tumor suppressor genes (*TP53*) cell differentiation (*SP1, GLI1*), and regulation of cell division (*HRAS*, *KRAS*). Overall, while multiple regulatory networks and pathways are shared across the species, there is also great variability when it comes to specific gene expression.

### LGR5 is expressed early in hair follicle development and throughout morphogenesis

**Figure 5:**
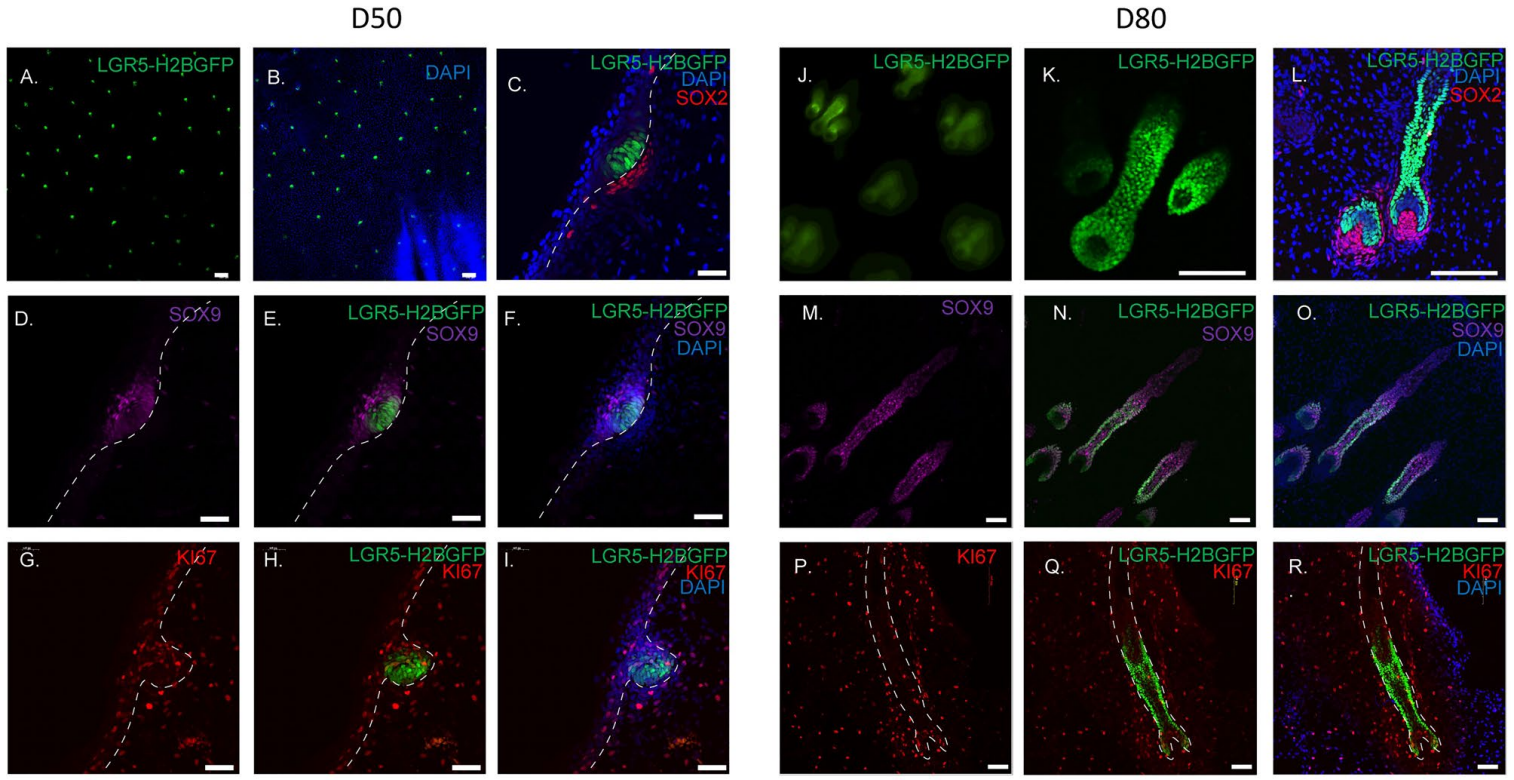
LGR5 is expressed early and late throughout developing porcine hair follicles. Fetal porcine skin at day 50 of gestation (**A**–**I**). (**A**, **B**) Whole mount confocal imaging showing patterning of LGR5 (**A**) and overlay with DAPI (**B**). (**C**) Cryosectioned immunostaining shows LGR5-H2BGFP cells throughout the hair germ, perpendicular to the basement membrane and adjacent to the developing dermal papillae marked by SOX2. Dashed lines demarcate the dermis/epidermis boundary and the location of the basal membrane. (**D**–**F**) Expression of SOX9 in relation to LGR5-H2BGFP showing LGR5 cells express low or no SOX9 in the placode. (**G**–**I**) Comparison of LGR5-H2BGFP and KI67 staining show little overlap, confirming our qPCR-data that the LGR5-H2BGFP cells are a slow-cycling population. (**J**–**R**) Day 80 porcine fetal skin. (**J**) Whole mount staining shows triplet pattern of follicular development. (**K**, **L**) LGR5-H2BGFP cells in the base of the follicle remain in close contact with the dermal papillae (SOX2) as they invaginate into the developing dermis. (**M**–**O**) LGR5-H2BGFP expression is found in the outer root sheath of the lower developing follicle, partially overlapping with SOX9 expression which is not limited to the outer root sheath. (**P**–**R**) KI67 shows that the few proliferating LGR5-H2BGFP cells (yellow) are found at the base of the hair follicle (dashed lines), adjacent to the dermal papillae. Scale bar represents 200 μM (**A**, **B**), 50 μM (**C**–**I**), or 100 μM (**K**–**R**).

We next asked how LGR5 expression fits in fetal hair follicle neogenesis. In pigs we examined two key fetal stages, gestational day 50 (D50) when hair follicles are in the late placode/early hair germ stage, and gestational day D80 (D80) in the follicle stage of morphogenesis. At D50, whole mount dorsal fetal skin shows the evenly patterned distribution of LGR5 expression throughout the epidermis (Fig. 5A,B), and LGR5-H2BGFP cells are oriented perpendicular and directly adjacent to the dermal condensate marked by SOX2 (Fig. 5C). SOX9, a known marker of hair follicle progenitor cells, occupies the suprabasal position and only dimly overlaps with LGR5 expression (Fig. 5D–F). The majority of LGR5-expressing cells are not proliferating, with only a few LGR5-H2BGFP cells in the suprabasal position co-expressing both KI67 and LGR5-H2BGFP (Fig. 5G–I), indicating that at this stage, LGR5-H2BGFP cells are slow cycling and that the primary proliferating population is in the suprabasal position. Previous reports in mice suggest there is a group of basal cells that are Wnt-high, Shh-high, and are the slow cycling progenitors that give rise to the Sox9+ population^35^. To prove that the mouse Shh population is equivalent to the LGR5-H2BGFP cells in pigs, we sorted LGR5-H2BGFP positive and LGR5-H2BGFP negative cells from D50 skin, evaluated *SHH* expression by RT-qPCR (Fig. S5A, B), and found that the LGR5-H2BGFP positive population is significantly enriched in *SHH* expression (Fig. S5C), confirming that LGR5 is expressed in the earliest stem cell progenitor population of the hair follicle.

At D80, during the follicle stage of morphogenesis, LGR5-H2BGFP expression is robust throughout the lower bulge (Fig. 5J, K). Interestingly, the pattern of follicle development in dorsal skin emerges as a triplet (Fig. 5J, K), with all developing follicles enveloping the dermal papillae, shown by SOX2 expression (Fig. 5L). While LGR5 specifically marks the developing outer root sheath of the lower follicle, SOX9 is expressed in mostly all cells in the lower follicle (Fig. 5M–O). The majority of proliferating LGR5-H2BGFP cells were found at the base of the hair follicle, close to the dermal papillae, similar to anagen (Fig. 5P–R).

**Figure 6:**
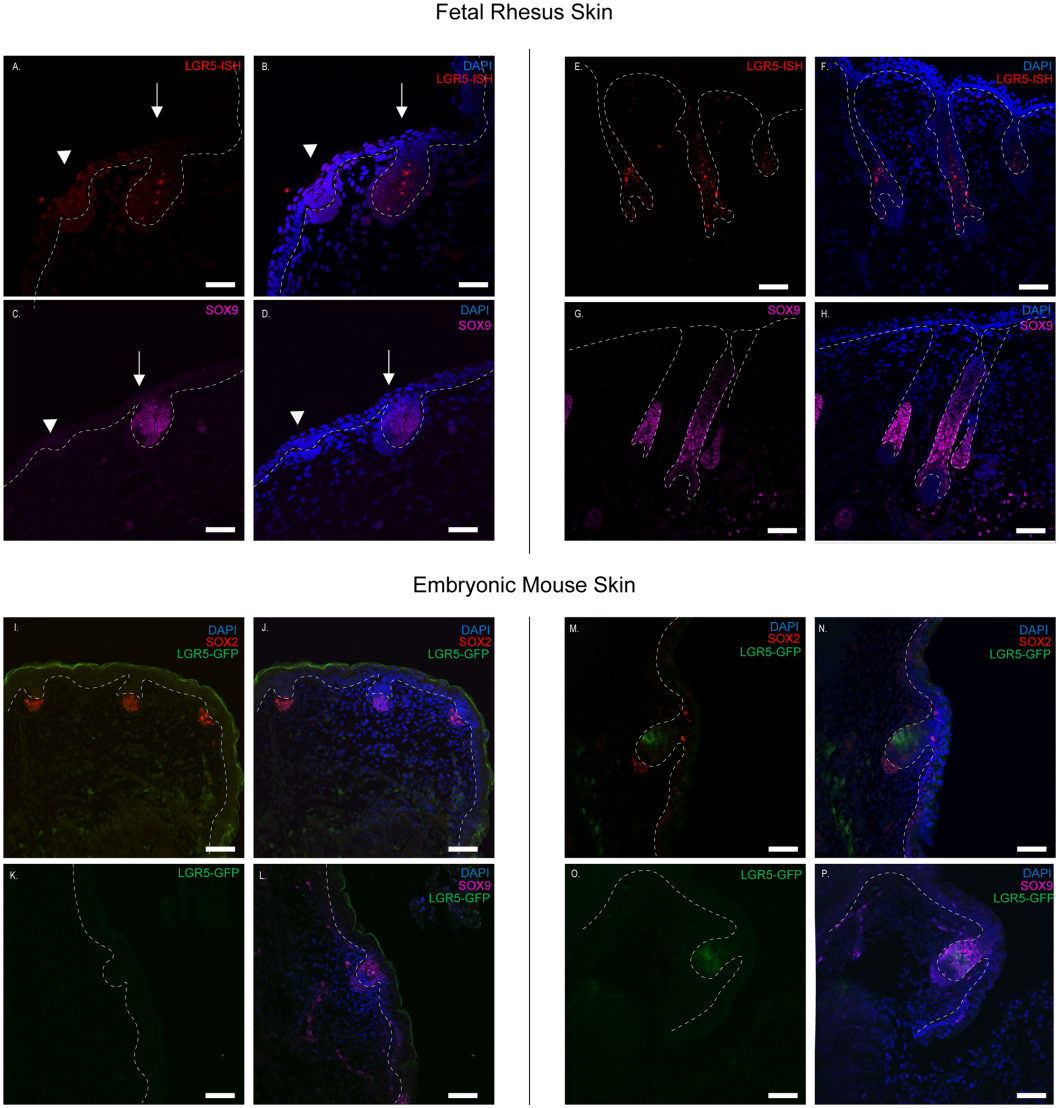
LGR5 expression in rhesus and mouse hair during follicle morphogenesis. In rhesus *LGR5* mRNA was detected using RNAscope (Red- *LGR5-ISH*) as detailed in the materials and methods section. Dashed lines delineate the border between the developing dermis and epidermis. (**A**–**D**) Gestational day 60 and (**E**–**H**) gestational day 90 samples. (**A**, **B**) LGR5 mRNA (Red- LGR5-ISH) is present, but expressed at a low level in the placode (arrow head), while it is robustly expressed in the hair peg (arrow). Similarly, SOX9 protein expression is absent in the placode (arrowhead), but present in the hair peg (arrow). The cells directly adjacent to the dermal papillae, with a perpendicular orientation to the basement membrane, are SOX9 negative. (**E**–**H**) Gestational day 90. As in pigs, the rhesus monkey hair follicles also develop in a triple pattern. (**E**, **F**) *LGR5* mRNA expression and (**G**, **H**) SOX9 protein expression is maintained throughout the lower hair follicle. (**I**–**P**) Day E15.5 hair follicle development in mouse tail (**I**–**L**) and dorsal skin (**M**–**P**). LGR5 expression was localized using a transgenic LGR5-eGFP line and anti-GFP antibodies. Embryonic day 15.5 mouse showing the placode (**I**–**L**) or peg stage (**M**–**P**) development, respectively. LGR5-GFP is undetectable or absent at the placode stage, although SOX2 (dermal papillae) and SOX9 (placode) are present. In the hair peg stage, LGR5-GFP is present, but limited to the mid-peg region and not in contact with the dermal papillae, similar to the pattern of SOX9 expression. Images are representative examples of at least three biological replicates per stage per species. Scale bars represents 50 μM.

To ask if LGR5 expression is conserved throughout development, and since human fetal skin was difficult to obtain, we obtained age-matched fetal rhesus skin and queried *LGR5* expression by RNA-FISH. *LGR5* is expressed at a low level in the placode (arrowhead), and strongly expressed in the hair peg (arrow) (Fig. 6A, B). SOX9 expression is absent from the placode, but is found throughout the hair peg (Fig. 6C, D). Interestingly, we consistently found that none of the cells in the hair peg, perpendicular to the basement membrane and adjacent to the dermal papillae, were SOX9 positive, although LGR5 was detected in these cells. Finally, LGR5-H2BGFP expression is consistent and robust throughout the lower hair follicle throughout development, along with SOX9 (Fig. 6E–H).

While previous studies have reported that LGR5 is not detected in mice until E18.5^8,36^, cryosections from *LGR5-eGFP* mouse dorsal skin show that LGR5eGFP is expressed as early as 15.5 (Fig. 6I–P). Since follicles develop at different stages based on location, we collected tail or dorsal skin for placode or hair germ stages, respectively. LGR5eGFP was undetectable at the placode stage (Fig. 6I, J), although SOX9-expressing cells occupied the same suprabasal position as in other species (Fig. 6K, L). In mice, we show that LGR5eGFP is first detectable in the hair peg stage (Fig. 6N–P), although the pattern of expression was more limited than the pig and rhesus skin, is not expressed in cells that contact the dermal papillae, and closely aligns with SOX9 expression. From these results, we can conclude that LGR5 is expressed early and throughout hair follicle development in all species examined, although expression in rhesus monkey and pig hair follicle morphogenesis is earlier and more widespread than in the mouse.

## Discussion

Many studies have attempted to find markers for HFSC, but few of these markers are conserved across species. Some studies have suggested that CD200 may be a shared marker for HFSCs between mice and humans^37,38^, however we and others show that CD200 in mice and pigs is expressed throughout the whole epidermis^6,7,39^. CD34, a functional marker of HFSC in mice, is not enriched in human HFSC^37^ as our results confirm in pigs. KRT15 has also been used as a marker of HFSC in mice, however it is also found in the entire basal layer of neonatal human skin, and in the rete ridges of adult human skin^40^. Despite the variation in patterns in many of the epidermal genes across species, in this work we show that in postnatal skin, LGR5 is a consistent marker of HFSC in the bulge across mice, pigs, and humans. In previous work in human cells, *LGR5* was detected in human hair stem cell organoids^41^, in the hair follicle region of haired men but not of bald men (alopecia)^42^, and was identified via single cell RNAseq in human skin as a potential marker for the hair follicle lower bulge^33^. Nevertheless, this work is the first to spatially define the position of *LGR5* expression in human hair follicles using RNA in situ hybridization.

The creation of the transgenic porcine model provides the opportunity to perform a cross-species examination of the transcriptome from the same cell type across multiple species. We recognize that our RNAseq analyses could not account for the differences in methods of collection from each dataset (especially related to depth of sequencing single cells vs bulk and number of replicates). However, it provides a starting point for examination of which genes and pathways are conserved, namely genes involved in extracellular matrix organization and extracellular matrix structure. More work needs to be done to understand the role of these stem cells in the modulation of the extracellular matrix and how that affects the cell fates within the niche, especially as hairs undergo cycling.

Beyond molecular comparisons of adult cells at homeostasis, we also can use this model to expand understanding of the role of LGR5 during follicle morphogenesis. The question has remained open as to what restricts canonical WNT signaling and polarizes the basal cells in epithelial bud formation in the placode^35^. Our results suggest that LGR5, a known potentiator of WNT signaling, could have a role in dictating which cells in the niche are WNT-responsive. Other studies have shown LGR5 to be involved with cell adhesion^43,44^, suggesting a role for LGR5 in anchoring asymmetrically dividing progenitors to the WNT-hi niche. While this could be true for rhesus and pig, LGR5 is not detected until after the placode stage in the mouse. Furthermore, when it is first detected, the pattern of expression in the mouse is similar to that of SOX9, and is not expressed in the WNT-hi cells adjacent to the dermal papillae. While the exact role of LGR5 in follicle morphogenesis is unclear, these differences in early morphogenesis could point to slightly different signaling pathways in early development between mice and other species.

Overall, the depth of knowledge that can be gained from an additional model beyond the mouse can provide more clues toward the behavior of stem cells across species through stages of development, homeostasis, and disease. The development of this *LGR5-H2B-GFP* transgenic pig represents a translational milestone in which we are able to both confirm and expand knowledge gained from mouse models and develop it toward human medicine. Future experiments using this model will enable us to study the mechanisms of how the hair follicle stem cells contribute to wound healing, improve our understanding of hair disease such as alopecia, and better elucidate the complexities of the hair follicle stem cell niche and understand the utility of these cells for skin and hair regeneration.

## Materials and methods

### Generation of *LGR5-H2B-GFP* pig

All experiments were approved by the Institutional Animal Care and Use Committee of North Carolina State University (IACUC protocol 17-028-B), and were performed in strict accordance with the IACUC-approved protocols in addition to the ARRIVE guidelines^45^. CRISPR/Cas9 nuclease was used to create a double-stranded break in the genomic DNA in exon 1 of the porcine LGR5 gene (gRNA sequence: ACCATGGACACCTCCTCGGT). The gRNA was selected to minimize any potential off-target effects using CRISPR RGEN and the Sus Scrofa genome. A homology-directed repair template plasmid containing H2B-GFP flanked by 1000 bp homology arms flanking the cut-site was co-transfected with the Cas9 (Gift from Keith Joung, Addgene #72247) and gRNA (Gift from Keith Joung, Addgene #43860) plasmids, and cells were seeded at low density for colony outgrowth.

Porcine fetal fibroblasts isolated from day 42 fetuses were used for gene editing and somatic cell nuclear transfer. After transfection and low density seeding for colony formation, colonies were genotyped by PCR and sequencing to verify successful targeted transgene integration before somatic cell nuclear transfer. Somatic cell nuclear transfer was completed as previously described^46^ and zygotes were surgically transferred into a surrogate and carried until term. Throughout this study, skin from 6 juvenile (2–4 months), 3 adult (> 6 months), and 3 fetal day 50 and 3 fetal day 80 pigs were used. The results shown are consistent across offspring derived from somatic cell nuclear transfer (F0) in addition to their progeny (F1) and are representative of both sexes. Moreover, breeding allows for segregation of any OTE, if present.

### Human, rhesus, and mouse samples

Human samples from adult male forearm skin were obtained from Accio Biobank Online and fixed in formalin within 24 h of death. Samples were collected in accordance with legal and ethical requirements of the United States. Samples were embedded in paraffin and sectioned at 7uM for further analyses.

Rhesus monkey fetal skin sections were kindly provided by Dr. Alice Tarantal, UC Davis. Two gestational ages were assessed: 60 days gestation (early second trimester) and 90 days gestation (late second trimester). Sections (5–6 um) were provided from formalin-fixed paraffin-embedded tissues and used for RNA-FISH and immunohistochemistry. Specimens were previously obtained under IACUC-approved protocols, and in accordance with the Animal Welfare Act and Public Health Service Policy on Human Care and Use of Laboratory Animals.

For all species at least three biological replicates per developmental stage were examined. Mouse samples were obtained after humane euthanasia from both male and female and a total of 4 adult Lgr5-EGFP-IRES-creERT2 transgenic mice (LGR5-eGFP) and at least 4 transgenic fetal mice.

### Immunofluorescence

Tissue was fixed with 4% paraformaldehyde then frozen in Optimal Cutting Temperature Compound (OCT) and sectioned at 20 µM. Sections were blocked with IHC/ICC Blocking Buffer (Invitrogen) with 0.4% Triton X-100 (Sigma), incubated with primary and secondary (1:5000) antibodies and finally mounted in Prolong Gold Antifade Mount with DAPI (ThermoFisher). Antibodies and dilutions Anti-Ki67, 1:100 (Abcam ab15580); Anti-KRT14 1:200 (Thermo Fisher MA1-06323); Anti-CD200, 1:50 (ls-b11638); AQ3 1:200 (Abcam ab125219); Anti-KRT10 1:200 (Abcam ab9025); Anti-KRT40, 1:100 (Abcam ab16113); Anti-KRT5, 1:100 (Abcam ab64081). Immunostained samples were visualized by confocal microscopy (Olympus Fluoview FV3000 Confocal Microscope)**.** Line profile intensity was measured using ImageJ (NIH).

### Single cell isolation and fluorescence activated cell sorting

Juvenile or adult porcine skin was cut into 5mm^2^ pieces and incubated with 10 mg/mL Dispase II (Sigma) in PBS without calcium and magnesium (Corning) for 1.5 h at 37 °C or overnight at 4 °C. Hair and epidermis were manually removed from dermis and incubated in 0.05% trypsin for 5–10 min at 37 °C with shaking. Suspension was vortexed and strained with 70 µm cell strainer (BD Falcon). Cells were resuspended in PBS with 10% fetal bovine serum (Corning) and 1% antibiotic–antimycotic (Corning). 250 ng/mL propidium iodide (Biotium) was added to cells for live/dead detection and samples were sorted by Beckman Coulter MoFlo XPD**.** Cells were sorted for GFP Hi, Lo, and Neg and data were further analyzed using FlowJo™ (BD Biosciences).

### Reverse transcriptase quantitative polymerase chain reaction (RT-qPCR)

Total RNA from skin tissue or sorted skin cells was isolated using Zymo Quick-RNA Microprep kit with on column DNase digest according to manufacturer’s instructions. RNA was eluted into DNase/RNase free water and stored at -80 °C until further use. cDNA was synthesized with AffinityScript Multiple Temperature cDNA Synthesis Kit (Agilent), according to the manufacturer instructions. For RT-qPCR, iTaq Universal SYBR Green Supermix (BioRad) was used with cDNA template and forward and reverse primers were designed as listed in Table S1. For optimal conditions: 2 min denaturation, 40 cycles of 95 °C denaturation for 5 s and 60 °C extension for 30 s, with final extension at 60 °C for 2 min. Primer sequences can be found in Figure S1. Each sample was amplified on a qTOWER^3^ thermal cycler (Analytik Jena) with technical duplicates for three biological replicates with similar results. Each gene expression was normalized to *GAPDH* and *ACTB.*

### RNA in situ hybridization

RNA in situ hybridization (RNA-FISH) was performed using RNAscope (Advanced Cell Diagnostics) according to the manufacturer's instructions. Briefly, paraffin embedded skin tissue was sectioned at 7um. Slides were deparaffinized with xylene, then heat treated followed by protease digestion. The tissue was hybridized with a 10 ZZ probe targeting either the 560–1589 region of Homo sapiens *LGR5* mRNA, the 466–1464 region of Sus scrofa *LGR5* mRNA, or the 494–1423 region of Macacca mulatta *LGR5* mRNA. As controls, a positive control probe was used against porcine (1–642 region) or human (139–989 region) cyclophilin B, Rhesus peptidyprolyl isomerase B (119–916 region), or negative control probes targeting the bacterial gene dapB were used, followed by chromogenic development. Slides were washed, then mounted with Prolong Gold Antifade Mount with DAPI (ThermoFisher) and imaged by confocal microscopy.

### Single-cell and bulk RNA-seq datasets and processing

We compiled two tissue-matched, single-cell epidermal RNA-seq datasets based on published samples for human, and mouse together with newly generated pig bulk RNA-seq samples. Aligned and processed sequencing data from single-cell human [accession numbers GSM3717037^33^] and mouse [GSE67602^34^] epidermal and hair follicle profiling studies were obtained from the Gene Expression Omnibus (Edgar, Domrachev, and Lash 2002). Single cell barcodes that had non-zero values for less than 500 genes or a high proportion of mitochondrial gene expression (> 5%) were excluded from further analysis. Bulk RNAseq samples were prepared from porcine cells, at least 500 ng of RNA was extracted from sorted LGR5-H2BGFP-high or LGR5-H2BGFP-negative populations from two pigs, same as prepared for RT-qPCR. RNAseq was performed externally by GENEWIZ; library preparation with poly(A) selection was performed followed by paired end 150 bp sequencing on Illumina HiSeq.

### Clustering and analysis of differential gene expression

Single-cell populations were clustered based on *LGR5* mRNA gene expression into either *LGR5* + (expression > 1) or *LGR5*- (expression = 0), while bulk RNA-seq data was clustered based on fluorescent markers. A student's t-test was used to calculate p-values for each species followed by a Benjamini–Hochberg multiple test correction at a false discovery rate of 0.05 (Soneson and Robinson 2018). Further core and comparative analyses of the differentially expressed genes were conducted using Ingenuity Pathway Analysis (IPA) along with gene ontology (GO) analysis using the ClusterProfiler R package (QIAGEN 2020; Krämer et al. 2014; Yu et al. 2012; Oliveros 2007).

## Supplementary Information

Supplementary Information.

## Data Availability

The RNA-sequencing data reported in this study have been deposited in NCBI’s Gene Expression Omnibus with the accession number GSE190069.
