# Supplementary material for: LGR5 is a conserved marker of hair follicle stem cells in multiple species and is present early and throughout follicle morphogenesis
Source: Sci Rep. 2022 Jun 1;12:9104. doi: 10.1038/s41598-022-13056-w (PMC9160037; doi:10.1038/s41598-022-13056-w)
Supplement: Supplementary file 1 — Supplementary Information. [file 41598_2022_13056_MOESM1_ESM.pdf]

Gene	F (5'-3')	R (5'-3')
ACTB	ACTGCCGCATCCTCTTCCTC	CTCCTGCTTGCTGATCCACATC
GAPDH	ATCCTGGGCTACACTGAGGAC	AAGTGGTCGTTGAGGGCAATG
LGR4	GACCGTCGGGTAGATTGCTC	CCAGCCAATCGTAGCTCCTC
LGR5	CCTTGGCCCTGAACAAAATA	ATTTCTTTCCCAGGGAGTGG
LGR6	CAGGAGGACGGCTTCATGC	GAGCTCCGTGAGGTTGTTCA
CD34	GGTATCTGCCTGGAGCGAAA	GGGTCTTCGCCCAGCCTTT
SOX9	CGGTTCGAGCAAGAATAAGC	GTAATCCGGGTGGTCCTTCT
KRT5	CGACAACGTCAAGAAGCAGT	GAGAGGGTGTTTGTGACGAC
KRT15	GCGAGATGGAGTGCCAGAAC	TCCACTGACTCCTCGACGTT
KRT14	GGAGGTGAAGATCCGCGAC	TCTGCAGCACGACATTAGCG
CD200	TGTTCCAAGTTACTAATCAGGCTGAA	AGCCCATTAGCAACATGATACTCTTT
SHH	CAGTTTATCCCCAACGTGGC	CCACTGGTTCATCACGGAGA
TCF4	TGCCTTAGGGACGGACAAAG	ATAGTTCCTGGACGGGCTTG
WNT3A	GCGACTTCCTCAAGGACAAG	GGTCACGTGTACCGAAGGAT
LRIG1	GACGCGGAGCCTAAACCTAA	CTCCACGCTGCGAATCCTAT
HOPX	GGAGGAGACCCAGAAATGGTT	TCTTGGTGGAAGGAAGCAGC
KI67	GGACCAGGCACAATGGATGG	CAGCTTTTGTCGAAGCGTCC

Table S1. Genes and primer sequences used in RT-qPCR analyses

Supplementary figures

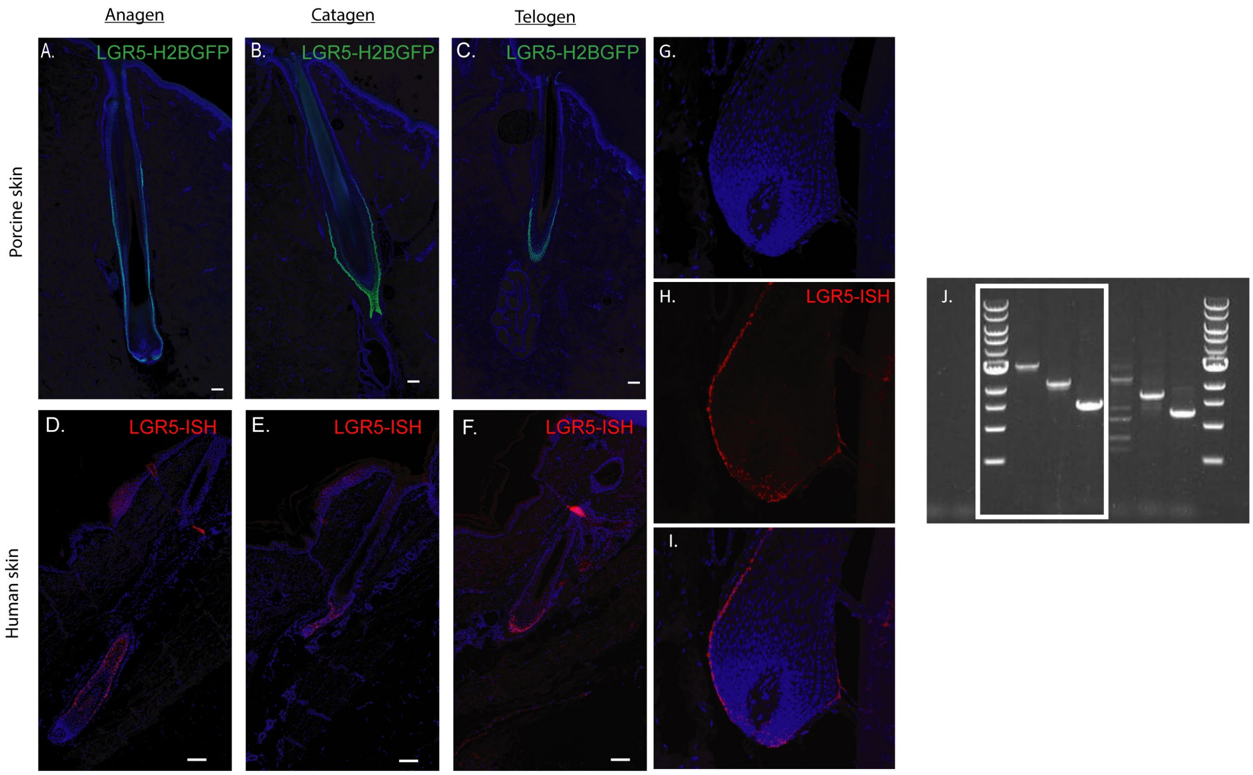

Supplementary Figure 1. Detection of LGR5-expressing cells by detection of LGR5-H2BGFP protein (pigs) or *LGR5* mRNA by situ hybridization (humans/*LGR5-ISH*) at different stages of the hair cycle. Expression of LGR5-H2BGFP in porcine anagen (A), catagen (B), and telogen (C) stage follicles. Detection of *LGR5* expression in human hair follicles by RNA *in situ* hybridization. As in pigs, *LGR5* is expressed in the outer root sheath of the lower bulge in anagen (D), catagen (E), and telogen (F) Scale bar represents 100 µM. (G-I) Higher magnification of porcine anagen-stage follicle showing *LGR5* mRNA expression using *LGR5-ISH*. Note reduced expression from the base of the follicle towards the hair shaft. Scale bar represents 100 µM. (J) Related to Figure 1B. The white frame shows where the image was cropped to generate Fig 1. The additional lanes contain samples from an unrelated experiment.

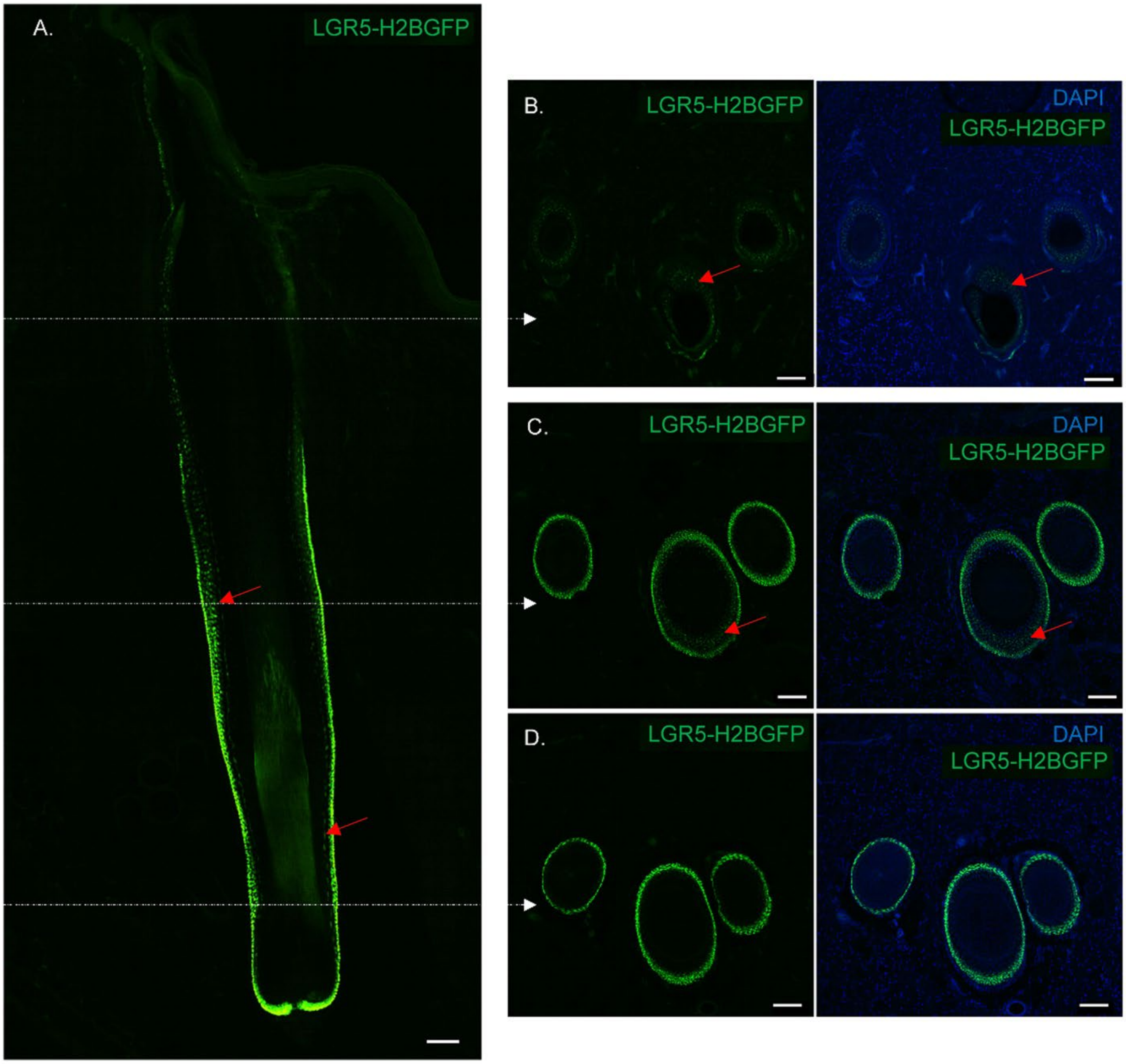

Supplementary Figure 2. Expression of LGR5-H2BGFP in the outer and inner shoot sheath along the length of the hair follicle. Cross section of porcine hair follicle shows that LGR5-H2BGFP is expressed at a high level in the outer root sheath, and low level in the inner root sheath (red arrow). A-D) Cross sections of hair follicles show distribution of LGR5-H2BGFP along the length of the follicle in correspondence with dashed white lines. Scale bar represents 200 μM.

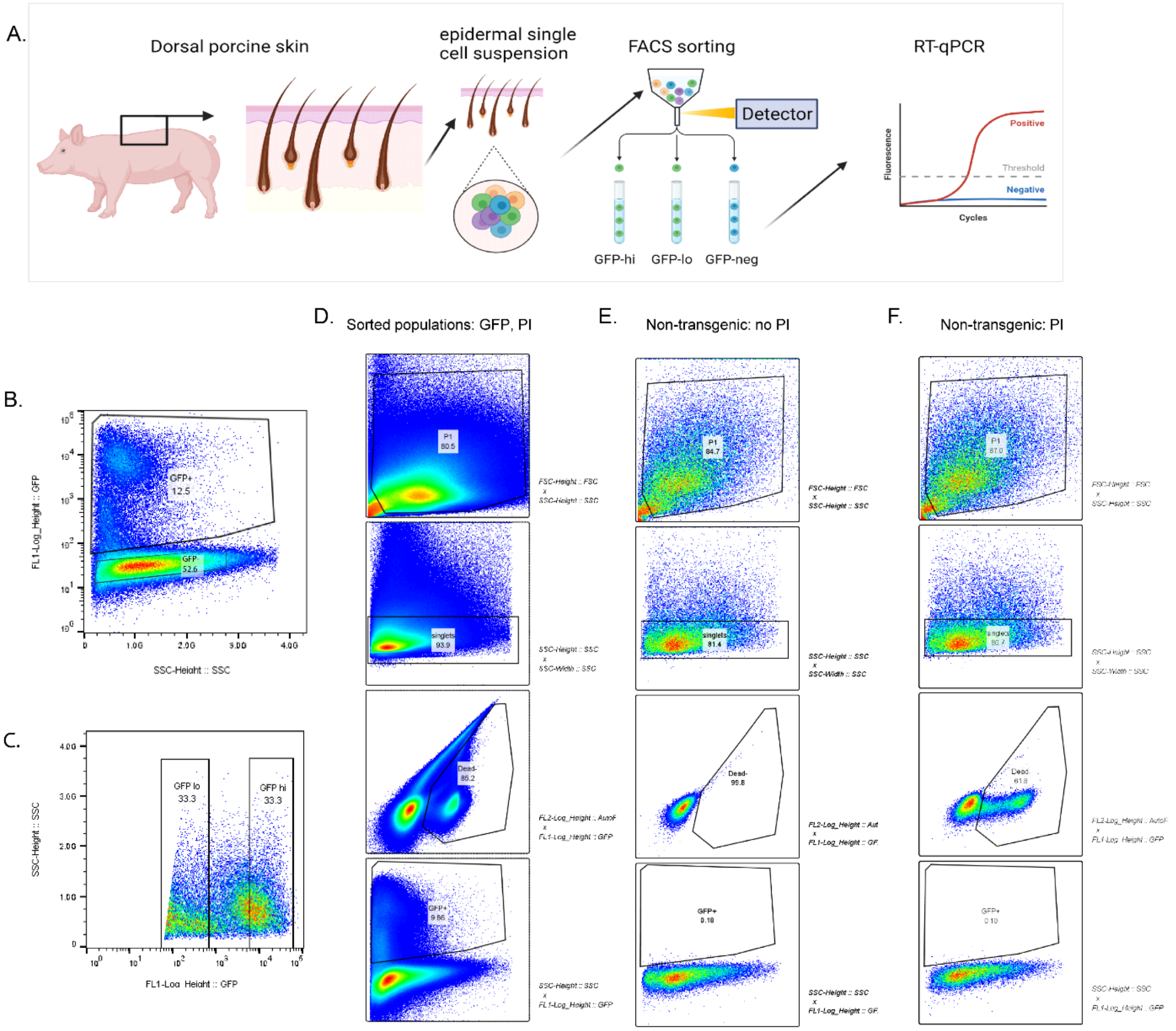

Supplementary Figure 3. Representative flow cytometry gating strategy and controls. A) Schematic depicting process of cell isolation and fluorescence activated cell sorting (FACS), created with BioRender. B) Cells were first sorted into negative and positive populations based on LGR5-H2BGFP expression. C) LGR5-H2BGFP positive cells were the split into H2BGFP-high or H2BGFP-low populations. Gating strategies determined as follows: D) Transgenic *LGR5-H2B-GFP* porcine epidermis stained with propidium iodide (PI) for live-dead, E) non-transgenic porcine epidermis with no PI, F) non-transgenic porcine epidermis with PI.

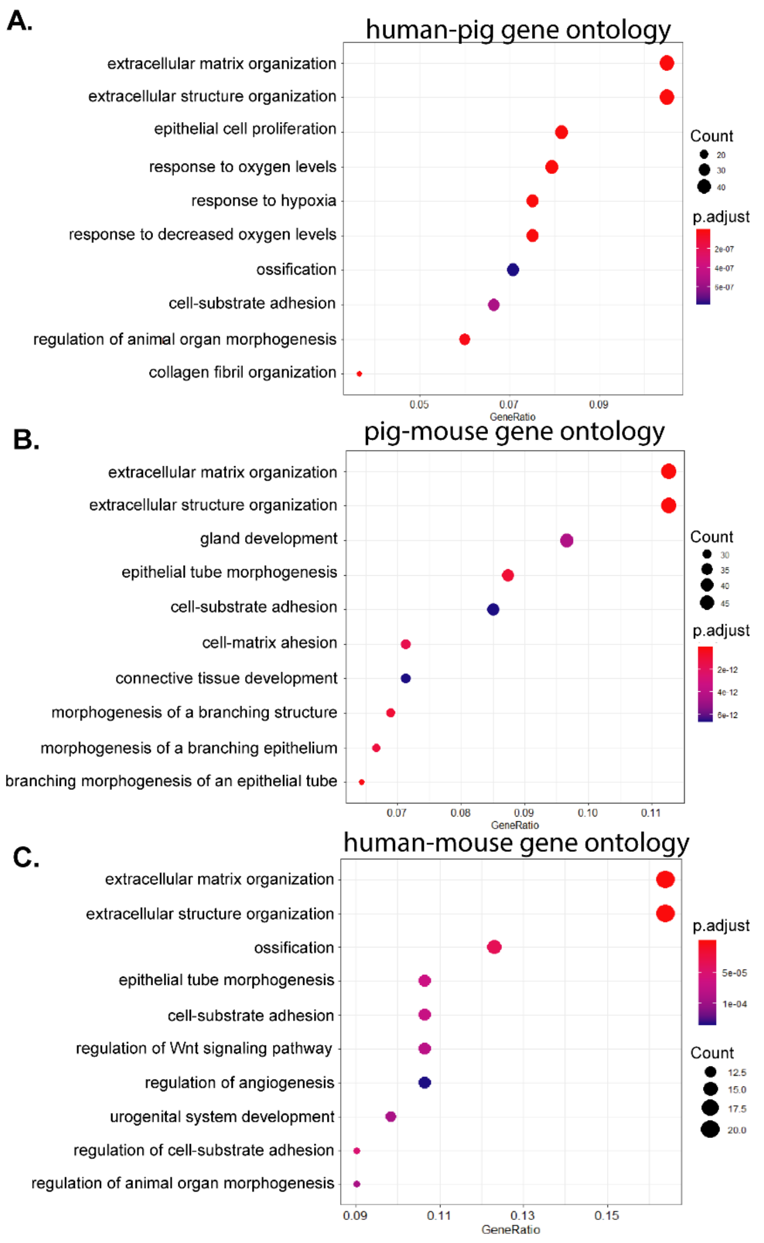

Supplementary Figure 4 (Related to main text Figure 4) Shared upregulated gene ontology pathways of upregulated genes in LGR5-high cells, compared pairwise across human, mouse and pig datasets. Species pair-wise comparison of significant upregulated gene ontology pathways showing each comparison shared many of the same impacted pathways in particular those related to extracellular matrix organization.

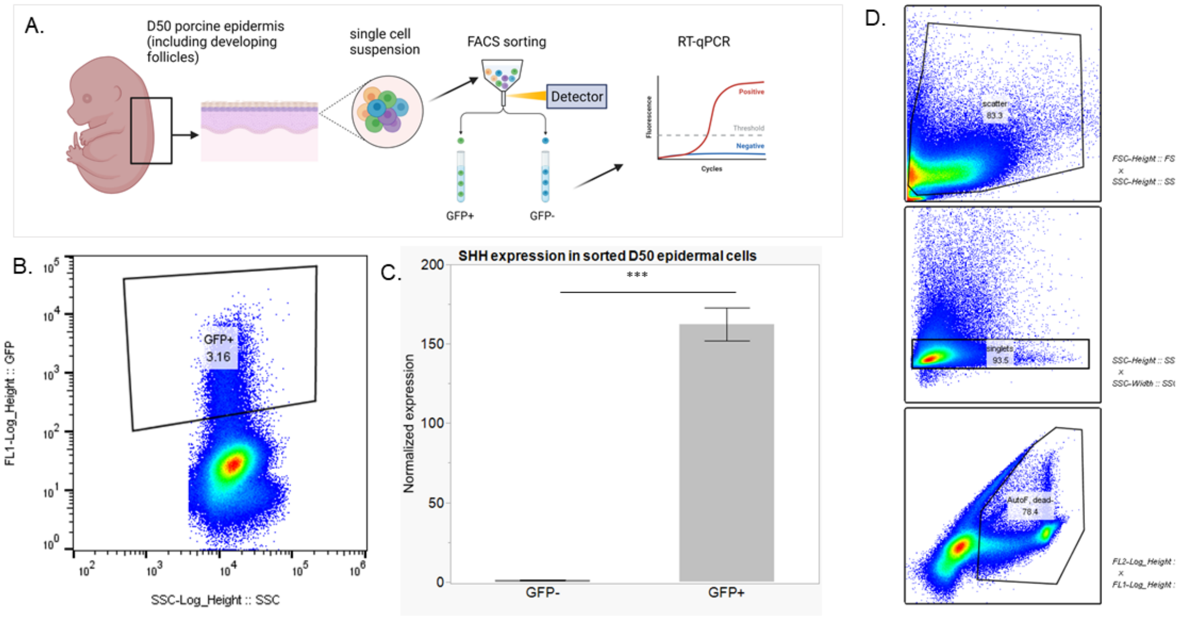

Supplementary Figure 5. ***SHH*** **expression in porcine LGR5-H2BGFP positive D50 epidermal cells.** A) Schematic depicting cell isolation, sorting, and RT-qPCR analysis processes, created with BioRender. B) Representative fluorescence activated cell sorting plot representing GFP+ population from a transgenic *LGR5-H2BGFP* fetus. C) RT-qPCR relative expression of *SHH* of LGR5-GFPH2B positive vs LGR5-H2BGFP negative sorted cells. Samples were normalized using a ddCT analysis to *GAPDH* and *ACTB* and then to the LGR5-H2BGFP negative sample. Student's t-test *** indicates P=0.02, n=2 pigs. D) Gating strategy reflecting panel B ancestry to gate on live singlets.
